# Detection of spotted fever group *Rickettsiae* in ticks from Zhejiang Province, China

**DOI:** 10.1007/s10493-015-9880-9

**Published:** 2015-01-30

**Authors:** Jimin Sun, Junfen Lin, Zhenyu Gong, Yue Chang, Xiaodong Ye, Shiping Gu, Weilong Pang, Chengwei Wang, Xiaohua Zheng, Juan Hou, Feng Ling, Xuguang Shi, Jianmin Jiang, Zhiping Chen, Huakun Lv, Chengliang Chai

**Affiliations:** 1 Zhejiang Provincial Center for Disease Control and Prevention, Hangzhou, China; 2 Taizhou Municipal Center for Disease Control and Prevention, Taizhou, China; 3 Jindong Center for Disease Control and Prevention, Jindong, China; 4 Anji Center for Disease Control and Prevention, Anji, China; 5 Tiantai Center for Disease Control and Prevention, Taizhou, China; 6 Daishan Center for Disease Control and Prevention, Daishan, China; 7 Xianju Center for Disease Control and Prevention, Xianju, China

**Keywords:** Spotted fever group *Rickettsiae*, Public health, *Rickettsia monacensis*, *Rickettsia heilongjiangensis*, *Rickettsia japonica*, Ticks

## Abstract

Tick species distribution and prevalence of spotted fever group *Rickettsiae* (SFGR) in ticks were investigated in Zhejiang Province, China in 2010 and 2011. PCR was used to detect SFGR and positive amplicons were sequenced, compared to published sequences and phylogenic analysis was performed using MEGA 4.0. A total of 292 adult ticks of ten species were captured and 7.5 % (22/292) of the ticks were PCR-positive for SFG *Rickettsia*. The PCR-positive rates were 5.5 % (6/110) for *Haemaphysalis longicornis,* 3.6 % (1/28) for *Amblyomma testudinarium* and 16 % (15/94) for *Ixodes sinensis,* respectively. Phylogenetic analyses of *gltA* genes detected in ticks indicated that there are two dominating groups of SFGR. Sequences of group one were closely related to *Rickettsia monacensis*, whereas sequences of group two were closest related to *Rickettsia heilongjiangensis* and *Rickettsia japonica*, which are human pathogens. Our findings underline the importance of these ticks in public health surveillance in Zhejiang Province, China.

## Introduction

*Rickettsia* is a genus of non-motile, gram-negative, intracellular bacteria transmitted by ticks, fleas, lice and mites and cause diseases in humans such as typhus, Rickettsial pox, Boutonneuse fever, African tick bite fever, Rocky Mountain spotted fever, Flinders Island spotted fever and Queensland tick typhus (Raoult and Roux 1997; Nathan et al. 2007). The genus *Rickettsia* is traditionally classified into the conventionally well-defined typhus group (TG), the spotted fever group (SFG), ancestral group and transitional group, based mainly on phenotypic and serological features (Gillespie et al. 2008). SFG *Rickettsiae* (SFGR) are widely distributed throughout the world in foci of endemicity and cause sporadic outbreaks in areas such as Japan, southern China and eastern Russia (Choi et al. 2005). From about 30 SFGR described so far, at least 15 are known to be pathogenic for humans (Parola et al. 2005).

In China, many SFGR belong to *Rickettsia sibirica* group, including two subspecies, i.e., *R. sibirica sibirica,* the agent of North Asian tick typhus detected in *Dermacentor silvarum* and *Dermacentor sinicus* in northern China, and *Rickettsia sibirica mongolitimonae,* the agent of lymphangitis-associated rickettsiosis isolated from *Hyalomma asiaticum* in Inner Mongolia (Yu et al. 1993; Zhang et al. 2006). *Rickettsia heilongjiangensis,* firstly isolated from *D. silvarum* ticks in Heilongjiang Province, can cause spotted fever in humans (Fournier et al. 2003; Jiao et al. 2005). Additionally, ticks are widely distributed in Zhejiang Province where humans are frequently bitten by ticks. This stimulated us to explore the tick species distribution in different areas of Zhejiang Province and the prevalence of SFGR species in these ticks.

## Materials and methods

### Tick sampling

**Fig. 1:**
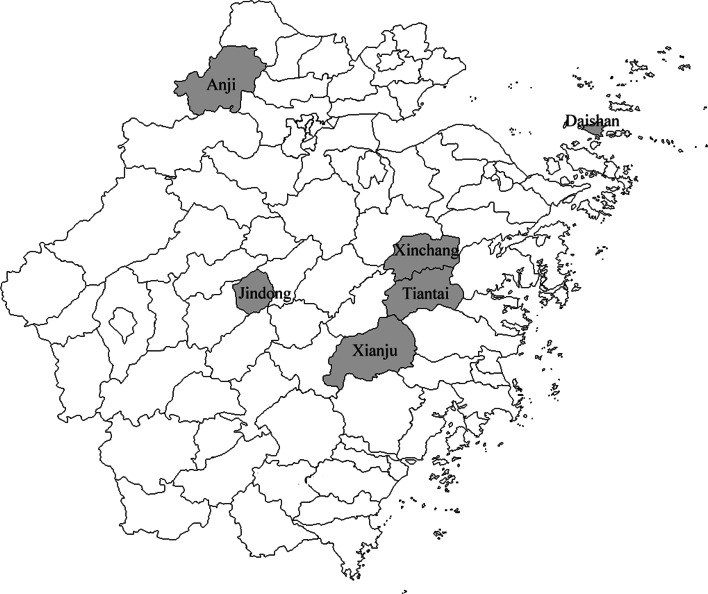
Geographical distribution of investigated sites in Zhejiang Province

The investigated sites included Daishan, Xinchang, Jindong, Tiantai, Xianju and Anji which were randomly chosen based on their geographical and administrative locations (Fig. 1). Ticks were collected from sheep, cattle, hedgehogs, domestic dogs, wild boar and small mammals including *Apodemus agrarius, Rattus niviventer,* and *Suneus murinus* during January 2010 to December 2011. All ticks were identified to the species level by standard guides (Deng and Jiang 1991) according to morphology and were stored at −80 °C prior to DNA extraction.

### DNA extraction

Each adult tick was subjected individually to DNA extraction. Ticks were washed using 70 % ethanol once; then they were washed three times with sterile deionized water to decontaminate the surface. Individual ticks were placed into different sterilized mortars and crushed with corresponding sterile pestles with liquid nitrogen. DNA was prepared from the crushed ticks using the QIAamp Tissue Kit (QIAGEN, Hilden, Germany) according to the manufacturer’s instructions.

### Polymerase chain reaction amplification

All tick samples were screened for SFGR infection through testing them individually by polymerase chain reaction (PCR) amplification with the use of primer (5′-GGGGGCCTGCTCACGGCGG-3′; 5′-ATTGCAAAAAGTACAGTGAACA-3′) designed to amplify a fragment of the citrate synthase gene (*gltA*) of SFGR as described previously (Regnery et al. 1991; Roux et al. 1997).

The reaction mixture contained 10 mM Tris–HCl, 1.5 mM MgCl_2_, 50 mM KCl (pH 8.3), 200 mM each dNTP, 1.25 U Taq polymerase, and 0.5 mM each respective primer. PCR products were electrophoresed in a 1.5 % agarose gel, stained with gold view, and visualized using ultraviolet light. To avoid cross contamination, all steps were performed in separate rooms; mastermix was prepared under a laminar air flow bench. In each PCR, at least two negative controls contained mastermix and sterile water instead of DNA template were used.

### Cloning and sequencing of PCR products

After electrophoresis, all positive DNA amplicons were purified using the Promega Wizard PCR Preps Kit (Promega, Madison, WI, USA) and then cloned into the PGEM-T Easy vector system (Promega) following the manufacturer’s protocol. The white recombinant clones were selected for sequencing. Bidirectional sequencing of positive PCR products were commercially conducted by Shanghai Sangon Biotechnology (Shanghai, China).

### Database DNA comparisons

Our sequences were submitted to GenBank and compared to published sequences using the BLAST program from the National Center for Biotechnology Information Website (http://www.ncbi.nlm.nih.gov/BLAST/), and phylogenic analysis was performed using MEGA 4.0. For each gene analyzed, a phylogram was constructed by the neighbor-joining method. Confidence values for individual branches of the resulting tree were determined by bootstrap analysis with 1,000 replicates.

### Data analysis

A Chi-square test or Fisher’s exact test, as appropriate, was used to compare SFGR prevalence among different species of ticks and different sampling sites. The difference was considered statistically significant when *P* < 0.05. Statistical analysis was performed with 16.0 (SPSS, Chicago, IL, USA).

## Results

### Tick samples

A total of 292 adult ticks of ten species were captured (Table 1). The species of ticks from different areas varied significantly (χ^2^ = 408.915, *P* < 0.001). The dominant tick species differed at various study areas. *Haemaphysalis longicornis* was dominant in Daishan (91.38 %) and Xinchang (80.00 %); *H. longicornis* (21.43 %), *Rhipicephalus haemaphysaloides* (21.43 %), and *Ixodes sinensis* (22.86 %) in Jindong; *I. sinensis* (100 %) in Tiantai; *H. longicornis* (58.14 %) and *Rhipicephalus microplus* (41.86 %) in Xianju; *Amblyomma testudinarium* (23.96 %) and *I. sinensis* (65.63 %) in Anji.

**Table 1:** Prevalence of spotted fever group *Rickettsiae* (SFGR) infection among tick species from different areas in Zhejiang Province, China

	Daishan	Xinchang	Jindong	Tiantai	Xianju	Anji	Total (n)	SFGR positive (n)	Positive rate (%)
*H. longicornis*	53	8	15	0	25	9	110	6	5.45
*R. haemaphysaloides*	3	2	15	0	0	0	20	0	0
*A. testudinarium*	0	0	5	0	0	23	28	1	3.57
*I. sinensis*	0	0	16	15	0	63	94	15	15.96
*R. microplus*	1	0	0	0	18	0	19	0	0
*I. granulatus*	1	0	0	0	0	0	1	0	0
*H. yeni*	0	0	5	0	0	0	5	0	0
*D. taiwanensis*	0	0	9	0	0	0	9	0	0
*H. hystricis*	0	0	5	0	0	0	5	0	0
*H. asiaticum*	0	0	0	0	0	1	1	0	0
Total (n)	58	10	70	15	43	96	292	22	7.53
SFGR positive (n)	1	1	7	0	4	9	22		
Positive rate (%)	1.72	10.00	10.00	0	9.30	9.38	7.53		

### Prevalence of SFGR infection

Overall, 7.53 % (22/292) of the ticks were PCR-positive to SFGR. The PCR-positive rates were 5.5 % (6/110) for *H. longicornis,* 3.8 % (1/28) for *A. testudinarium,* and 16 % (15/94) for *I. sinensis,* respectively (Table 1). No *Rickettsia* DNA was detected in *R. haemaphysaloides, R. microplus, I. granulates, Haemaphysalis yeni, D. taiwanensis, Haemaphysalis hystricis,* and *Haemaphysalis asiaticum.* The prevalence of *Rickettsia* was not significantly different among species (χ^2^ = 15.776, *P* = 0.072).

Prevalences in Daishan, Xinchang, Jindong, Tiantai, Xianju, and Anji were 1.7, 10, 10, 0, 9.3, 9.4, and 7.5 %, respectively. The prevalence was similar among different areas (χ^2^ = 5.391, *P* = 0.37).

### Phylogenetic analysis

**Fig. 2:**
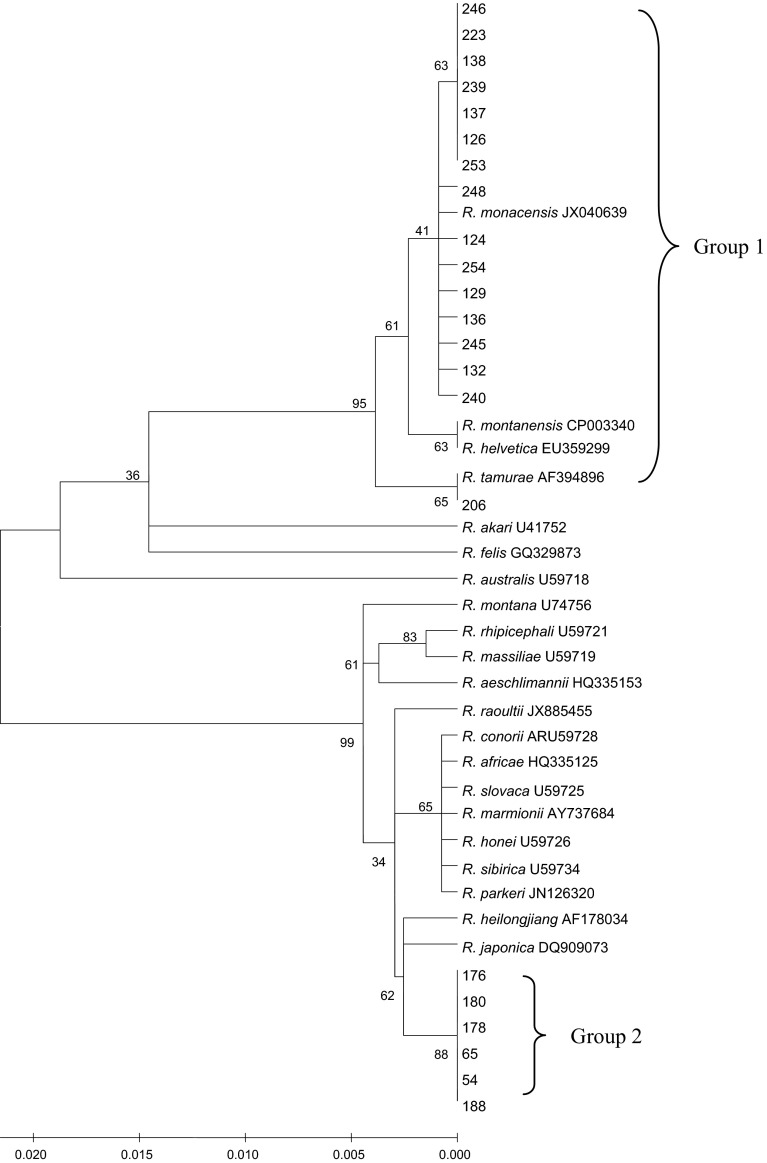
Phylogenetic analyses of partial *gltA* genes of *Rickettsia* species identified in ticks from Zhejiang Province, China

All sequences were submitted to GenBank, and the accession numbers were from KM886876 to KM886897. Phylogenetic analyses of *gltA* genes (340 bp) detected in ticks indicated that there were two dominating groups of SFGR (Fig. 2).

Group one consisted of 16 detected sequences, which were from Anji (n = 9) and Jindong (n = 7). Moreover, all sequences were detected in *I. sinensis* except that one sequence was detected in *A. testudinarium.* The majority of sequences of this group were closely related to *R. monacensis* (JX040639), which was detected in *I. ricinus* ticks from Romania. One sequence was most similar to *R. tamurae* (AF394896) that came from *A. testudinarium* in Japan.

Group two included six detected sequences, which were from three areas (Daishan, Xianju and Xinchang). Of note, these sequences were all from *H. longicornis* and were most similar to *R. heilongjiangensis* (AF178034) and *R. japonica* (DQ909073).

## Discussion

In our study, ten species of ticks were found in six areas and the tick species varied significantly at different areas. This may be due to the different animal hosts from which they were removed, geographic sites, unbalanced number of ticks in different areas. The positive rate of SFGR was found to be 7.5 % among ticks, a percentage similar to the recently reported percentage (6.9 %) recorded in the Hebei Province, China (Zou et al. 2011). However, ticks were pooled prior to DNA extraction and the pools consisted of 2–10 ticks collected from the same site in their study.

Although no SFGR DNA was detected in Tiantai and infection rates varied at different survey areas, we could not determine the geographic diversity of SFGR in ticks. The number of ticks examined was limited and the species of ticks varied significantly; therefore, infection rates in the current study might be biased. In addition, because intensity of circulation of any vector-borne agent fluctuates dramatically throughout the year and from year to year, even at the same location (Bown et al. 2003; Wielinga et al. 2006), we could not justify comparing infection rates between different areas on the basis of unsynchronized single collections over a 2-year period. A randomized sampling scheme and further collection of ticks are required to clarify this issue.

Usually, for SFGR, ticks are both the reservoir and the vector of the bacteria and the geographical distribution of the disease is superimposed upon that of the tick (Raoult and Roux 1997). Among ten tick species collected in this study, only three were infected with SFGR. This demonstrated that these three tick species were the main carriers of SFGR. But it is not excluded that other ticks might act as vectors for SFGR because of small sample sizes.

Phylogenetic analyses indicated that two groups of SFGR were detected in ticks, one identified in Jindong and Anji, another identified in Xinchang, Daishan and Xianju. Interestingly, sequences of group 1 were all from *I. sinensis* and *A. testudinarium,* sequences of group two were all from *H. longicornis.* Additionally, one sequence which was detected in *A. testudinarium* was most similar to one sequence, which was also detected in *A. testudinarium* in Japan. These indicate that SFGR species might be related to tick species.

Some sequences were most similar to *R. monacensis* which had been isolated from *I. ricinus* in Germany (Simser et al. 2002) and Hungary (Sreter et al. 2005). *Rickettsia monacensis* can cause spotted fever in humans, as the disease attributed to this agent has not received a specific name. The other sequences were most related to *R. heilongjiangensis* and *R. japonica* which were also human rickettsial pathogens. *Rickettsia heilongjiangensis* was first isolated in 1982 from *D. silvarum* in Heilongjiang Province in the northeast of China and subsequently demonstrated to cause human spotted fever in China and in the Russian Far East (Mediannikov et al. 2004). *Rickettsia japonica* was first isolated in 1984 in Japan (Mahara 1997) and is the agent of Japanese spotted fever (Uchida et al. 1992).

In comparable studies throughout Zhejiang Province, SFGR were detected in *H. longicornis, R. haemaphysaloides* (Cheng et al. 2010; Meng et al. 2008; Jiang et al. 2010). However, sequences detected in their study were most similar to *R. rhipicephali* and *R. massiliae.* This is the first identification of *R. monacensis* in ticks from Zhejiang Province which demonstrate the importance of epidemiological survey of *R. monacensis* infection in Zhejiang Province.

There were several limitations to our study. Firstly, the low numbers of ticks of each species in each region collected in different times and from different animals reduced the probabilities of getting useful information from this study. Secondly, the examined ticks were collected from animals rather than as questing specimens which brings into question whether SFGR would survive moulting of the ticks, especially for non-*Ixodes* ticks. Finally, tick species from different sites varied significantly, which may also influence infection rates of different sites.

In summary, we detected SFGR in diverse species of ticks from different areas suggesting that SFGR is widely prevalent in ticks in Zhejiang Province. SFGR detected were similar to *R. monacensis, R. heilongjiangensis* or *R. japonica* which can cause human infection. SFGR infections are largely unrecognized but may become more frequently diagnosed in Zhejiang Province.
